# Antifungal Activity of *Beauveria bassiana* Endophyte against *Botrytis cinerea* in Two *Solanaceae* Crops

**DOI:** 10.3390/microorganisms8010065

**Published:** 2019-12-31

**Authors:** Lorena Barra-Bucarei, Andrés France Iglesias, Macarena Gerding González, Gonzalo Silva Aguayo, Jorge Carrasco-Fernández, Jean Franco Castro, Javiera Ortiz Campos

**Affiliations:** 1 Instituto de Investigaciones Agropecuarias (INIA) Quilamapu, Av. Vicente Méndez 515, Chillán 3800062, Chile; afrance@inia.cl (A.F.I.); jorge.carrascof@inia.cl (J.C.-F.); jean.castro@inia.cl (J.F.C.); javiera.ortiz@endofitos.com (J.O.C.); 2 Facultad de Agronomía, Universidad de Concepción, Vicente Mendez 595, Chillán 3812120, Chile; mgerding@udec.cl (M.G.G.); gosilva@udec.cl (G.S.A.)

**Keywords:** entomopathogenic fungi, biological control, antifungal activity, *Solanum lycopersicum*, *Capsicum annuum*

## Abstract

*Botrytis cinerea* causes substantial losses in tomato and chili pepper crops worldwide. Endophytes have shown the potential for the biological control of diseases. The colonization ability of native endophyte strains of *Beauveria bassiana* and their antifungal effect against *B. cinerea* were evaluated in *Solanaceae* crops. Root drenching with *B. bassiana* was applied, and endophytic colonization capacity in roots, stems, and leaves was determined. The antagonistic activity was evaluated using in vitro dual culture and also plants by drenching the endophyte on the root and by pathogen inoculation in the leaves. Ten native strains were endophytes of tomato, and eight were endophytes of chili pepper. All strains showed significant in vitro antagonism against *B. cinerea* (30–36%). A high antifungal effect was observed, and strains RGM547 and RGM644 showed the lowest percentage of the surface affected by the pathogen. Native strains of *B. bassiana* colonized tomato and chili pepper tissues and provided important levels of antagonism against *B. cinerea.*

## 1. Introduction

Among the phytosanitary problems affecting crops in the *Solanaceae* family, such as tomato (*Solanum lycopersicum* L.) and chili pepper (*Capsicum annuum* L.), fungal diseases that produce economic losses are highlighted. Intensive production systems, like greenhouse cultivation, high temperatures, and humidity, favor disease development and dissemination [1]. *Botrytis cinerea* is the etiological agent of grey mold, which affects more than 200 plant species and is the most frequent disease in the cultivation of tomato and chili pepper worldwide. This fungus can penetrate wounds and colonize the whole aerial part of the plant [2], affecting the whole growing cycle and postharvest [1] and reducing fruit yield and quality. In greenhouse conditions, *B. cinerea* enters the tissue through pruning cuts, leaves, and flowers. In turn, damage to the fruits occurs largely at postharvest [2,3].

*Botrytis cinerea* is difficult to control because it can infect using different strategies and use different hosts as its inoculum source because it can survive as mycelia, conidia, and/or sclerotia for long periods of time in crop residues and in the soil. Chemical fungicides are intensively used for its control; however, there is scientific evidence that highlights their detrimental effects on human health, the environment, and the economy [4,5]. Moreover, their effectiveness has been affected by the appearance of resistant strains [6]. In this context, the biological control of diseases using microbial agents has advantages over chemical fungicides, given the availability of agents such as fungi, yeasts, and bacteria [2] and their safety and decreased probability of producing resistant strains.

In recent years, world markets have shown a growing tendency towards the use of biological control agents as an alternative to chemically synthesized pesticides [7]. Many microorganisms are currently being used as biopesticides because they offer a series of additional benefits over and above their objective function [8]. Entomopathogenic fungi stand out in this group and have been extensively studied for their effects as epiphytes for use in pest control and, on a smaller scale, against plant diseases [9,10]. However, in the last few years, there has been also a growing interest to study them as endophytes.

Endophytic fungi are defined as microorganisms that spend most or all of their lifecycle colonizing host plant tissues without causing any apparent damage to the host [11]. They are associated with the majority of plants, found naturally in the ecosystem, and considered an extremely important partner for plant development [12,13]. These microorganisms have been of interest in recent years because of the beneficial characteristics they confer to their hosts [14]. Some of these benefits are plant growth promotion, inhibition of pathogenic organisms, removal of soil contaminants, and increased tolerance to extreme temperature, water availability, and salinity conditions [15,16,17], all of which are important features for future agrifood production.

Several studies have demonstrated that endophytic fungi can protect host plants against pathogens and herbivores [18,19,20,21,22]. The host plant receives multiple benefits from the interaction with the endophyte in exchange for carbon-based resources [23]. Endophytes can remain in plant tissues for long periods of their lifecycle, thus protecting them from pathogen attacks as well as potential environmental changes that could threaten their survival and biocontrol efficiency [24]. They can establish interspecific interactions, and the protection against pathogen attack is produced by direct mechanisms, such as competition, parasitism, and antibiosis (production of primary and secondary metabolites, enzymes, or volatile compounds), and indirect mechanisms, such as induction of resistance [18,25]. 

In the present study, we used native strains of the entomopathogenic fungus *Beauveria bassiana* to determine its ability to endophytically colonize tomato and chili pepper plants. We also investigated if its presence inside the plants enables the mitigation of the negative effects caused by *B. cinerea.*

## 2. Materials and Methods 

### 2.1. Source of Fungal Strains and Seed

Native strains of *B. bassiana* were used in the assays, isolated by the methodology described by France et al. [26], and identified by morphological and molecular methods. *Botrytis cinerea* strains isolated from tomato were also used. All strains were obtained from the Chilean Collection of Microbial Genetic Resources (CChRGM) (Table 1). Seeds of tomato var. Limachino—INIA traditional cultivar and chili pepper STa_01 used in this study, were obtained from the Sweet Saten Company.

### 2.2. Surface Seed Disinfection

Seeds were disinfected according to the protocol adapted from Ownley et al. [21] and Griffin [20]. Seeds were submerged in a 95% ethanol solution for 1 min and then in 1.5% NaOCl for 3 min. They were washed three times for 1 min in sterile distilled water. Seeds were dried at room temperature on sterile absorbent paper for 3 h in a biosecurity cabinet and then used in all the assays involving plants. To test seed disinfection, a water sample from the third wash was taken with a bacteriological loop and streaked onto a Petri dish with potato dextrose agar (PDA) medium. On another dish with the same medium, the surface of one of the disinfected seeds was printed to rule out epiphytic colonization. 

### 2.3. Plant Inoculation with B. bassiana Strains and Endophytic Colonization

Single conidia were inoculated on Petri dishes with 100% potato dextrose agar (PDA, Difco^TM^) and 150 mg L^−1^ chloramphenicol. The dishes were incubated in the dark at 25 ± 2 °C for 10 days. Conidia were harvested, and their viability was determined according to the methodology described by Moore et al. [27]. They were suspended in test tubes with 10 mL sterile distilled water and 0.01% (v/v) Tween 80% (Difco^Tm^), and dilutions were performed until the concentration of 1 × 10^6^ conidia mL^−1^ was reached. In the case of the *B. cinerea* pathogen, it was used at concentrations of 1 × 10^5^ conidia mL^−1^. The concentration of conidia was determined with a Neubauer counting chamber (BOECO, Germany), and the same inoculum was used for all the experiments.

Conidia suspension was inoculated in 100 mL test tubes containing 15 mL PDA [20]. One test tube was left without fungal inocula, as a control. Test tubes were incubated in the dark at 25 ± 2 °C for 4 days. Once the apparent growth of the fungus in each tube was observed, 20 mL of the substrate was added, which consisted of a mixture of perlite, peat, compost, and vermiculite (2:2:2:1) sterilized twice in an autoclave at 120 °C and 115 psi for 1 h. Controls were incubated at 25 ± 2 °C for 5 days in the dark. Two seeds were sown in each test tube, and after emergence, one plant per tube was left. The tubes were incubated for 30 days in growth chambers at 25 ± 2 °C with 12 h/12 h light/darkness photoperiod and arranged in a randomized design with six replicates.

### 2.4. Assessment of Endophytic Colonization

To assess the endophytic colonization of *B. bassiana* strains, plants were extracted from the tubes and washed with tap water, and cuts were made to separate roots, stems, and leaves (*n* = 5). Each tissue was disinfected with 70% ethanol for 2 min and 1.5% NaOCl for 5 min and rinsed three times for 1 min with sterile distilled water. Then they were left to dry on sterile absorbent paper, a modification of Resquín-Moreno et al. [28]. To control disinfection, the same procedure used to test the seeds was conducted. Once disinfection was accomplished, 10 subsamples were cut from each plant part (roots, stem, and leaves), resulting in a total of 30 pieces per plant. Root and stem samples were 10 mm long, whereas leaves were cut in 6 mm discs. The cuttings were distributed in Petri dishes with Noble agar (Difco™) medium plus chloramphenicol and incubated in the dark at 25 ± 2 °C for 30 days. After the incubation, the pieces were evaluated by the presence of fungus growing from the border of the tissues, and the result obtained was the percentage of endophytic colonization (PEC). Strains that exhibited no endophytic colonization were discarded for use in the following evaluations.

### 2.5. Morphological and Molecular Identification of Reisolate Strain

Agar samples (1 cm^2^) with mycelia growing from inside the leaves inoculated with the endophyte were placed on a slide. Fungal structures were observed under an optical microscope with 40× magnification and were identified through taxonomic keys [29]. For molecular identification, samples were taken from mycelia coming out of the plant tissues and sown in the PDA medium. The polymerase chain reaction (PCR) method was used with specific markers for *B. bassiana* P1 (5′AAGCTTCGACATGGTCTG) and P3 (5′GGAGGTGGTGAGGTTCTGTT) according to the methodology described by Hegedus and Khachatourians [30]. Amplification products were separated in agarose gel and observed under ultraviolet light. Once the identity was confirmed, monoxenic samples were taken from the reisolation of each strain and used as the inoculum in the next assays.

### 2.6. In Vitro Antifungal Activity of Endophytic B. bassiana

Mycelium discs (3 days, 5 mm diameter) from pure *B. bassiana* cultures obtained from plant tissues were placed 1.5 cm from the edge of a 9 cm Petri dish containing the PDA medium (20 mL). After 2 d, discs with *B. cinerea* pathogen (3 days, 5 mm diameter) were equidistantly placed opposite to the endophyte and incubated in the dark at 25 ± 2 °C for 10 d. Six replicates were included per treatment and were placed in a completely randomized design. At the same time, control dishes containing only the pathogen were prepared [31]. When the pathogen colonized the whole Petri dish, the percentage of radial growth inhibition of the pathogen (PRGIP) was determined by Formula (1). The radii of the colonies were measured (mm) in each of the treatments using a digital caliper.

(1) PRGIP:[(R1−R2)/R1] × 100

where R1 is the radius of the pathogen colony growing alone (mm) and R2 is the radius of the pathogen colony competing against the endophyte (mm). 

### 2.7. Evaluation of Antifungal Activity in the Host Plant

Previously disinfected tomato and chili pepper seeds were sown in individual 300 mL pots on a substrate consisting of a mixture of perlite, peat, compost, and vermiculite (2:2:2:1) sterilized twice in an autoclave at 120 °C and 115 psi for 1 h. Pots were left in growth chambers (at 25 ± 2 °C, 65% relative humidity, and 12 h/12 h light/darkness photoperiod) for 30 and 60 days for tomato and chili pepper, respectively. Plants were observed and watered with 3 mL of sterile distilled water every 5 days. Plants with 4–5 true leaves were inoculated with the endophyte by drenching the substrate with a solution of 5 mL sterile distilled water and 2 mL of the conidial solution (1 × 10^6^ conidia mL^−1^). The five strains that exhibited the best antifungal performance in the in vitro tests were selected. Pots were covered at their bases with aluminum foil to prevent cross-contamination of the endophyte and maintained for another 10 days in the growth chambers under the same conditions previously described. A conidial solution of the pathogen (1 × 10^5^ conidia mL^−1^) was then prepared, using a modification of the method by Martin-Hernández et al. [32], and applied 14 days after the endophytes as a foliar spray on tomato and chili pepper leaves (of the same age). For the analyses, three and two leaves located in the middle part of the chili pepper (eight weeks) and tomato plant (six weeks), respectively, were selected. The treatments were arranged in a randomized design, with five replications for each strain.

The disease incidence in both hosts was evaluated 10 days postinoculation of the pathogen [33] by calculating the percentage of the surface affected by the pathogen (PSAP) by Formula (2). The total area (TA) and affected area (AA) per leaf were obtained with the ImageJ, an open-source image processing software [34]. The software allows quantitatively determining the leaf area covered by sporulation, damage, or chlorotic and necrotic symptoms. For both species, the assay was conducted two times under the same conditions.

(2) PSAP:(S2/S1) × 100

where S1 is the total leaf surface, and S2 is the leaf surface affected by the pathogen. 

### 2.8. Statistical Analyses

In all cases, a completely randomized design was used. Prior to the statistical analyses, data were used to determine the normality and homogeneity of the variance. The results were used to perform variance analyses and the means were compared with Fisher’s least significant difference (F-LSD) test (*p* < 0.05) with the software InfoStat Version 2011 [35].

## 3. Results

### 3.1. Endophytic Colonization of B. bassiana Strains in Tomato and Chili Pepper Plants

**Figure 1:**
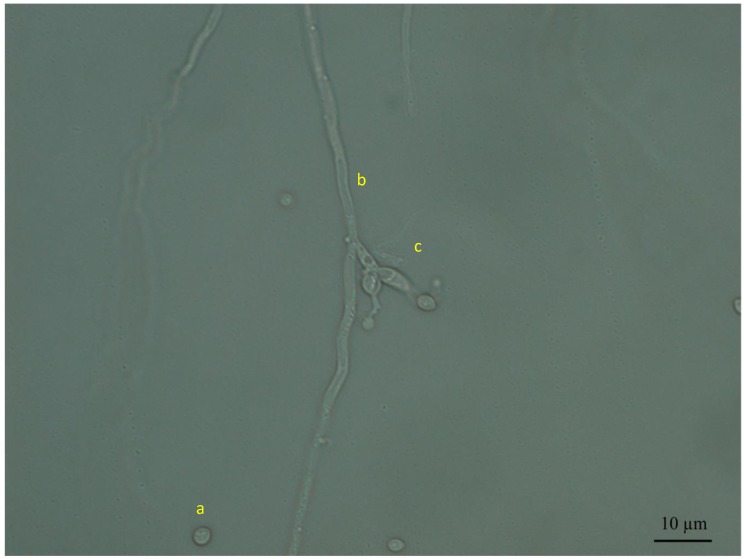
Morphological characteristics of endophyte *Beauveria bassiana* (RGM 644) on Noble agar (100×). (**a**) Conidia, (**b**) conidiogenous cells, and (**c**) hyphae.

The ten evaluated strains of *B. bassiana* colonized internally the different parts of the tomato plants, while eight strains colonized the chili pepper plants. White mycelium growing from the different plant tissues was observed with an optical microscope (Figure 1). Structures such as hyphae, conidiophore, and conidia of *B. bassiana* were observed in the different treatments and confirmed by morphology and subsequent molecular analyses. The specific molecular markers gave positive amplification in all of the evaluated strains; consequently, the endophyte identity was confirmed as *B. bassiana.*

By using both disinfection control methods (water and tissue printing), epiphytic colonization could not be detected, and therefore, all the observed mycelium growth came from the internal tissues. No *B. bassiana* was reisolated from the different tissue plants used as a control without fungal inoculation (Table 2). 

In tomato, 100% of the evaluated strains exhibited some degree of endophytic colonization. Colonization fluctuated between 10% and 48% in the leaves and 8% and 46% in the stem and roots. Furthermore, 100% of the strains demonstrated a systemic mode of action, where the fungus inoculated in the roots was re-isolated from the leaves. The strain with the best performance, considering the sum of all plant tissue, was RGM 557 (50%), while the strain RGM 393 had the worst performance (10%) (*F* = 1.24; df = 10; *p* = 0.29). For chili pepper, endophyte colonization fluctuated between 0–68% in the leaves, 0–14% in the stem, and 0–34% in the roots. Strains RGM 393 and RGM 461 exhibited no endophytic colonization ability. The strain with the best overall performance was RGM 547 with 35% (sum of all tissue plant colonized), and the worst performance (*F* = 14,76; df = 10; *p* < 0.0001) among the strains that achieved some degree of colonization was RGM 632 with 7%. The highest percentages of endophytic colonization between the different tissues of tomato plant were in stems. In the chili pepper plant, the highest percentages were in the leaves.

### 3.2. In Vitro Growth Inhibition of the Pathogen 

**Figure 2:**
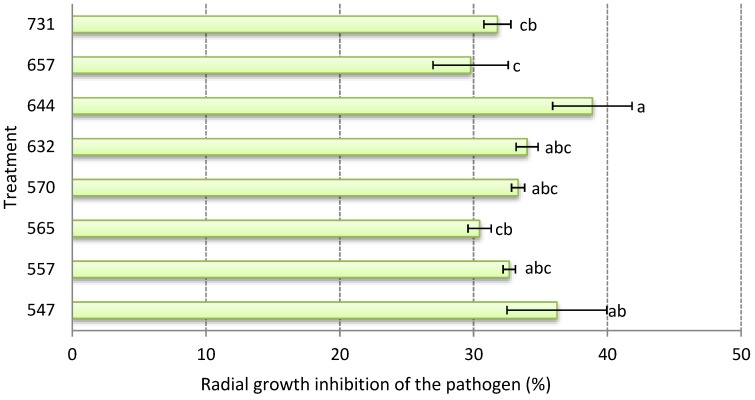
Radial inhibition growth of *Botrytis cinerea* (%) against strains of endophytic *Beauveria bassiana* at 7 days (*n* = 6). Data represent the mean ± standard error. Different letters over the bars represent significant differences among the treatments according to the Fisher’s LSD test (*p* < 0.05).

The monoxenic culture of the pathogen was able to totally colonize the Petri dish after 7 days in a 75 mm radius. When the advance of the pathogen against the endophytic strains was measured, all the cases showed a level of radial growth inhibition of the pathogen, and the PRGIP fluctuated between 30% and 39%. The pathogen was only able to grow 45 mm against strain RGM 644 (*F* = 2.13; df = 7; *p* = 0.062), while the greatest advance (53 mm) was against the strain RGM 657 (Figure 2).

**Figure 3:**
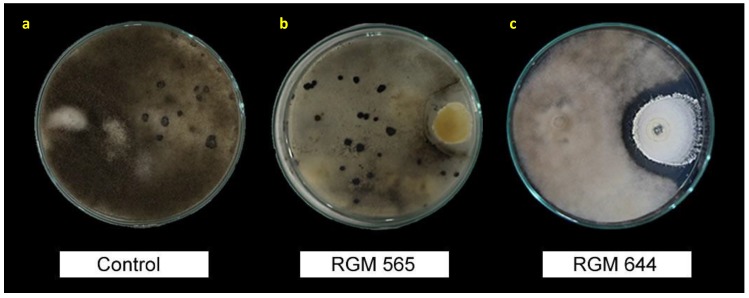
Dual cultures of *Botrytis cinerea* against different endophytic strains of *Beauveria bassiana*. (**a**) *Botrytis cinerea* (control) 7 d after inoculation, (**b**) pathogen against endophytic strains RGM 565 and (**c**) RGM 644, showing different mycelia density and inhibition of sclerotia.

It was observed that some strains did not allow mycelia to achieve the density and tonality found in the control plate. Moreover, in strains with the best performance in terms of PGIP, almost no sclerotia were detected (Figure 3).

### 3.3. Antifungal Activity in the Host Plant 

**Figure 4:**
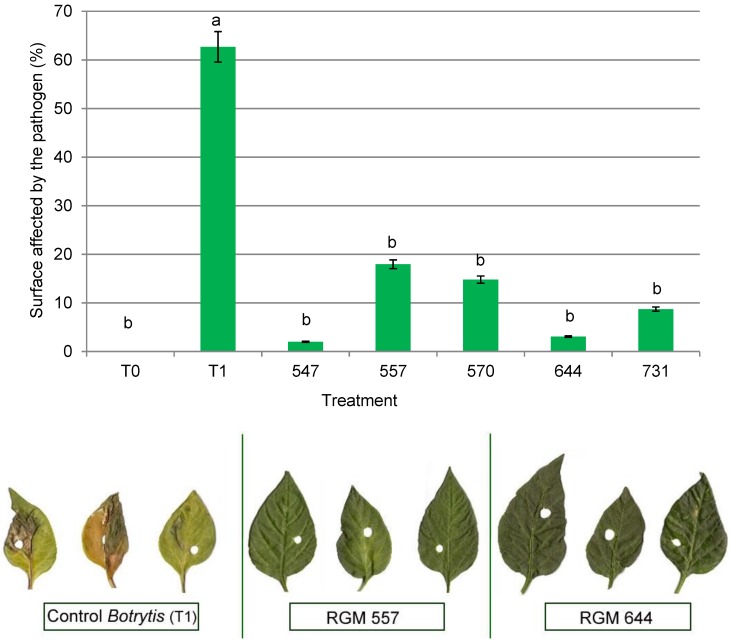
Surface affected by the pathogen (cm^2^) in chili pepper leaves 10 days postinoculation (*n* = 5). The leaves of plants not inoculated with *Botrytis cinerea* (T0) were asymptomatic. The leaves with endophytic strains showed a low level of symptoms (RGM 547, RGM 557, RGM 570, RGM 644, and RGM 731), while the leaves of plants inoculated with *B. cinerea* RGM 2519 (T1) exhibited chlorotic and necrotic spots. Values are expressed as means ± standard error. Means with different letters are significantly different at *p* < 0.05 by Fisher’s LSD test.

The PSAP in chili pepper leaves was noteworthy as being lower in plants (*F* = 11.46; df = 6; *p* < 0.0001) inoculated with endophytes compared to plants only inoculated with the pathogen. The plants inoculated with endophytes ranged from 2% to 18% of PSAP, with the lowest for RGM 547 strain and the highest for RGM 557. Meanwhile, treatment only with the pathogen exhibited early symptoms of the disease (during the first week of inoculation) with a PSAP of 63%, while some symptoms were observed after the second week in plants with endophytes (all endophyte treatment). Leaves of plants that were not inoculated with the pathogen showed no symptoms of the disease (Figure 4).

**Figure 5:**
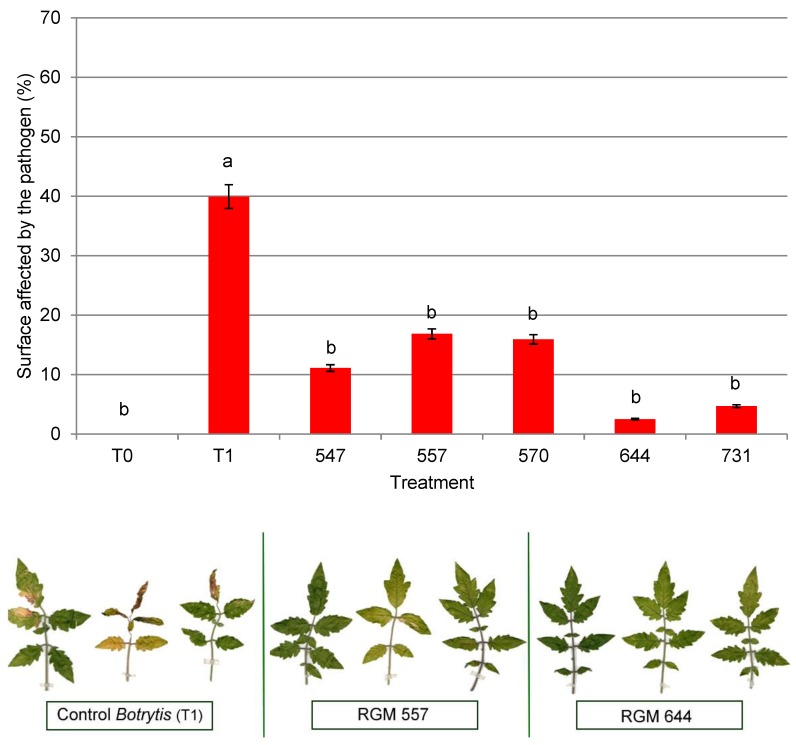
Surface affected by the pathogen (cm^2^) in tomato leaves at 10 days postinoculation (*n* = 5). The leaves of plants not inoculated with *Botrytis cinerea* (T0) were asymptomatic. The leaves with endophytic strains showed a low level of symptoms (RGM 547, RGM 557, RGM 570, RGM 644, and RGM 731), while the leaves of plants inoculated with *B. cinerea* RGM 2519 (T1) exhibited chlorotic and necrotic spots. Values are expressed as means ± standard error. Means with different letters are significantly different at *p* < 0.05 by Fisher’s LSD test.

In the case of tomato, leaves of plants inoculated with the pathogen had the highest PSAP (40.0%), which was notably higher (*F*  = 4.51; df = 6; *p* = 0.0007) than the percentages obtained for plants inoculated with endophytes. This percentage is similar to the result obtained in chili pepper. The range of PSAP for plants with endophytes was 2.5% to 16.9%, and significant differences existed among strains. The strains with the lowest percentage were RGM 644 and 731, with values of 2.5% and 4.7%, respectively. The strains with the highest pathogen incidence were RGM 557 (16.9%) and 570 (15.9%). Leaves of plants that were not inoculated with the pathogen exhibited no symptoms of the disease (Figure 5). 

## 4. Discussion

### 4.1. Endophytic Colonization of Beauveria bassiana Strains

The present study reports on the colonization of *B. bassiana* in tomato and chili pepper as endophytes. Every evaluated strain was able to endophytically colonize tomato plants; these results concur with those shown by other studies in tomato [20,21,36]. As for chili pepper, 80% of the strains endophytically colonized the plants. Studies conducted by Paul et al. [37] reveal an assorted diversity of endophytic microorganisms in chili pepper, including some entomopathogens as *Paecilomyces*, *Cordyceps*, *Cladosporium*, and *Penicillium*; however, the authors do not report the natural presence of *B. bassiana*. Recent studies on chili and sweet pepper demonstrated the endophytic colonization of *B. bassiana*, and these are some examples of the limited numbers of reports about the colonization on this species [38,39].

The results of endophytic colonization in tomato leaves (10–48%) in this study were lower than those presented by Klieber and Reineke [40], who obtained the highest colonization (60%). This may be due to several factors, such as the disinfection technique, which could kill the endophyte [36], the inoculation techniques [21], the type of leaf used for sampling (tissue age and location in the plant), the time between inoculation and reisolation [40], and the plant’s endophytic microbial community because actinobacteria and yeasts were also found in the reisolation. Several studies have provided evidence of the endophytic colonization of *B. bassiana* in plants inoculated by different methods through the seeds, leaves, and roots [21,41,42,43]. This fungus has been reported as a facultative endophyte of several plants [44], suggesting that *B. bassiana* is not a specific endophyte for a plant species or particular cultivar. This characteristic can be related to the specificity exhibited by entomopathogenic fungi with the host insect; this can be very limited in the case of obligate pathogens or very broad in the case of facultative pathogens [45]. 

It was observed that *B. bassiana* showed a systemic colonization pattern for both plant species, and thus, the fungus was reisolated from the roots, stem, and leaves. Previous studies conducted with other *Solanaceae* have also demonstrated a systemic colonization pattern for these fungi [39,43,46]. Behie et al. [47], in *Phaseolus vulgaris*, demonstrated that *B. bassiana* is able to colonize plant tissues on and below the soil, while endophytes of the genus *Metarhizium* prefer to colonize the roots. This colonization pattern is very interesting from the viewpoint of the development of biocontrollers because it would allow access to places in the plant that most chemical products cannot reach. 

The important colonization levels obtained for both species in the present assay can be related to the evolution of these fungi over time. Barelli et al. [48] suggest that some entomopathogenic fungi evolved from fungi related to plants (symbionts) and that the pathogenicity in insects is part of a more recent adaptive process. In other words, these fungi were endophytes at first and then were able to become independent of the plants and survive as insect pathogens. The same authors also propose that these fungi have never left their symbiotic relationship with the plant and that pathogenicity towards insects could be a strategy by which the endophytes have access to nitrogen sources in exchange for carbohydrates provided by the plant; this mechanism was reported by Herre et al. [23] and Behie [49].

Although there is an important number of reports about the endophytic colonization of *B. bassiana* and its action as a biological control agent, most of them have been carried out under controlled conditions, like the present study. However, field conditions could affect its action [50]. *Beauveria bassiana* is classified as saprophytic and exhibits poor competition in the soil [51,52]. Therefore, soil inoculations could be inefficient because of the poor survival of the endophyte when encountering more competitive microbiota, hindering its arrival to the roots and limiting its endophytic colonization [53]. On the other hand, in foliar applications, these fungi spend a significant amount of time on the leaf surface, and their germination could be affected by radiation, temperature, and humidity conditions [54].

### 4.2. In Vitro Growth Inhibition of the Pathogen Botrytis cinerea 

The important levels of pathogen inhibition by these nine strains of *B. bassiana* could be due to the ability of these fungi to produce a great variety of bioactive metabolites, which have antimicrobial properties [55,56]. Oosporein, beauvericin, bassianolide, bassianin, beauveriolide, bassiacridin, and cyclosporine are highlighted among the metabolites produced by this fungus [57,58,59,60], and of these, oosporein and beauvericin have antifungal activity [61,62]. Studies conducted by Feng et al. [63] determined that the genome of *B. bassiana* has at least 45 different groups of secondary metabolite biosynthesis gene clusters. The dissemination of these compounds in the medium affects the growth of pathogenic fungi. Other in vitro evaluations have also demonstrated the antifungal effect of the endophyte against pathogens such as *Botrytis cinerea*, *Cladosporium herbarum* [64], *Fusarium* spp. [64,65], *Gaeumannomyces graminis* var. *tritici* [66], and *Rhizoctonia solani* [67]. Antibiosis and/or competition are highlighted among the mechanisms used by the endophyte to inhibit pathogen growth. Mycoparasitism was not detected when observing the advance zone under the microscope; this suggests that the evaluated strains have no ability to parasitize *B. cinerea* hyphae.

### 4.3. Antifungal Activity in the Host Plant against Botrytis cinerea

The results obtained in this study suggest that the action of *B. bassiana* as an endophyte increase the plant’s ability to resist the attack of pathogens such as *B. cinerea.* Thus, the endophyte exhibits a relevant antifungal activity against *B. cinerea* in both tomato and chili pepper. *Beauveria bassiana* was applied to and subsequently colonized roots, moving up to the stem, presumably through the vascular system [55], and reaching the leaves where the pathogen was inoculated. Several plants inoculated with different strains were asymptomatic, while the control was affected, and PSAP was greater than 39%. A growing number of studies provide evidence of the protective action that *B. bassiana* confers on different pathogens [21,67,68,69,70]; the present study is the first report that demonstrates the protective effect of *B. bassiana* endophyte against *B. cinerea* on chili pepper and tomato.

The lowest PSAP obtained in chili pepper and tomato leaves could be the result of a direct or indirect effect of the endophyte in the plant. Direct effects would occur when the endophyte is able to enter through the roots, move through the vascular system until reaching the leaf tissues, and compete for space and food with the pathogen, reducing its colonizing ability [67]. Another possibility could be when pathogen hyphae enter the leaves and are parasitized by the endophyte hyphae (mycoparasitism), which weaken and decrease their damage potential [21].

The indirect effect could firstly be due to the action of secondary metabolites, as mentioned for dual cultures that could act in distant plant tissues from which they are produced. There would be enzymes degrading the cell wall within these metabolites, which is an important mechanism involved in controlling phytopathogenic fungi [70]. Secondly, indirect action could occur by activating the systemic resistance in the plant, an action mechanism used by the endophyte *B. bassiana* against zucchini yellow mosaic virus in pumpkin and against *Xanthomonas axonopodis* pv. *malvacearum* in cotton, as reported by Jaber and Salem [71] and Ownley et al. [21], respectively. According to Vega et al. [68] and Ownley et al. [16], the antifungal action exhibited by endophytes could be due to a mixture of the previously mentioned mechanisms rather than to the action of a single mechanism. 

## 5. Conclusions

The results of the present study provide evidence of the potential exhibited by endophytic strains of *Beauveria bassiana* to control *Botrytis cinerea* in chili pepper and tomato; eventually, they could be used to control diseases in other species of the *Solanaceae* family. Future research should focus on conducting assays under field conditions because of the possible effects of the environment on soil and leaf inoculations with the endophyte. It is also necessary to perform complete sequencing of the strains, aimed at identifying possible genetic relationships with their antifungal activity.

The use of endophytic fungi could overcome some of the challenges faced in controlling plant diseases such as chemical fungicide toxicity, the appearance of resistance of some pathogens, and food safety. The studied isolates represent excellent candidates for the development of biocontrol tools to control not only insects but also pathogens. They could be used preventively within an integrated management strategy due to the diversity of mechanisms by which they could act against the attack of different pathogens. However, as *Beauveria bassiana* produces a considerable amount of secondary metabolites that could act as mycotoxins, it is necessary to increase the number of studies to determine the potential damage that mycotoxins of this fungus could cause in health to people and animals in its action as endophytes.

## Figures and Tables

**Table 1:** Fungal strains used in this study.

Code Strain *	Species	Collection Location	Origin
RGM 393	*Beauveria* *bassiana*	Robinson Crusoe, Valparaíso Region, Chile.	Native forest soil
RGM 461	*Beauveria* *bassiana*	Cañete, Biobío Region, Chile.	Natural pasture soil
RGM 547	*Beauveria* *bassiana*	Santa Bárbara, Biobío Region, Chile.	Natural pasture soil
RGM 557	*Beauveria* *bassiana*	Los Lagos, Los Lagos Region, Chile.	Natural pasture soil
RGM 565	*Beauveria* *bassiana*	Portezuelo, Biobío Region, Chile.	Natural pasture soil
RGM 570	*Beauveria* *bassiana*	Molina, Maule Region, Chile.	Arable soil, *Vitis vinifera* fruit crop
RGM 632	*Beauveria* *bassiana*	Pencahue, Maule Region, Chile.	Natural pasture soil
RGM 644	*Beauveria* *bassiana*	Icalma, La Araucanía Region, Chile.	Natural pasture soil
RGM 657	*Beauveria* *bassiana*	Puerto Ibañez, Aysén del General Carlos Ibáñez del Campo Region, Chile.	Natural pasture soil
RGM 731	*Beauveria* *bassiana*	Río Cisnes, Aysén del General Carlos Ibáñez del Campo Region, Chile.	Natural pasture soil
RGM 2519	*Botrytis cinerea*	Colín, Maule Region, Chile	Tomato plant

* Accession number of microorganisms from the Chilean Collection of Microbial Genetic Resources—CChRGM.

**Table 2:** Endophytic colonization (%) of *Beauveria bassiana* in tomato and chili pepper (*n* = 5).

Species	Plant Part	Treatments										
		RGM 393	RGM 461	RGM 547	RGM 557	RGM 565	RGM 570	RGM 632	RGM 644	RGM 657	RGM 731	Control ^1^
Chili pepper	Leaves	0 ^2^ ± 0.0 ^3^ d ^4^	0 ± 0.0 d	68 ± 13.6 a	28 ± 13.6 bc	36 ± 16.0 b	36 ± 7.5 b	10 ± 3,2 cd	36 ± 7.5 b	32 ± 8.0 bc	52 ± 8.0 ab	0 ± 0.0 d
	Stems	0 ± 0.0 b	0 ± 0.0 b	2 ± 2.0 b	12 ± 2 a	14 ± 2.5 a	14 ± 2.5 a	2 ± 2.0 b	14 ± 4.0 a	14 ± 2.5 a	14 ± 2.5 a	0 ± 0.0 b
	Roots	0 ± 0.0 d	0 ± 0.0 d	34 ± 6.8 a	18 ± 5.8 bc	18 ± 8 bc	18 ± 3.7 bc	10 ± 3.2 cd	16 ± 5.1 bc	24 ± 9.8 abc	28 ± 3.7 ab	0 ± 0.0 d
Tomato	Leaves	10 ± 6.3 ab	10 ± 3.9 ab	34 ± 17.2 ab	48 ± 23.1 a	24 ± 19.4 ab	22 ± 11.8 ab	20 ±12.3 ab	24 ± 19.4 ab	26 ± 3.7 ab	18 ± 8.0 ab	0 ± 0.0 b
	Stems	12 ± 8.0 abc	36 ± 19.8 abc	44 ± 21.1 ab	38 ± 19.4 ab	36 ± 16. abc	46 ± 6.9 a	12 ± 8.0 abc	12 ± 8.0 abc	8 ± 5.8 bc	36 ± 6.0 abc	0 ± 0.0 b
	Roots	8 ± 4.9 b	18 ± 11.9 ab	24 ± 19.4 ab	46 ± 22.3 a	34 ± 17.2 ab	16 ± 5.1 ab	20 ± 12.3 ab	28 ± 18.6 ab	18 ± 9.7 ab	8 ± 4.9 b	0 ± 0.0 b

^1^ Control represents plants without *Beauveria bassiana* applications. ^2^ Mean values of endophytic (%) colonization (*n* = 6). ^3^ Standard error. ^4^ Mean values of the same treatment followed by a different letter are significantly different according to Fisher’s least significant difference (LSD) test (*p* < 0.05).
